# Response of Soil Microbial Diversity to Triple-Cropping System in Paddy Fields in Middle Reaches of Yangtze River

**DOI:** 10.3390/plants14091292

**Published:** 2025-04-24

**Authors:** Haiying Tang, Junlin Zhou, Ning Liu, Yao Huang, Qin Liu, Faizah Amer Altihani, Binjuan Yang

**Affiliations:** 1 School of Agriculture and Biotechnology, Hunan University of Humanities, Science and Technology, Loudi 417000, China; zjl1223179534@163.com; 2 Key Laboratory of Crop Physiology, Ecology and Genetic Breeding, Ecological Science Research Center, Jiangxi Agricultural University, Nanchang 330045, China; 15979908856@163.com (N.L.); huangyao202103@163.com (Y.H.); 13247712832@163.com (Q.L.); 3 Department of Biology, College of Science, King Khalid University, Abha 61413, Saudi Arabia; ftehany@kku.edu.sa

**Keywords:** soil microbes, nutrient cycling, paddy field, planting patterns, microbial diversity

## Abstract

To explore the characteristics of soil microbial community structure diversity for different planting patterns in paddy fields, and to screen out the planting patterns suitable for the promotion of double-cropping rice areas in the middle reaches of the Yangtze River, five typical planting patterns were set up in this study. The five patterns are Chinese milk vetch–early rice–late rice (CRR, CK), Chinese milk vetch–early rice–sweet potato || late soybean (CRI), rapeseed–early rice–late rice (RRR), rapeseed–early rice–sweet potato || late soybean (RRI) and potato–early rice–late rice (PRR). The variation characteristics of soil microbial community structure diversity and the correlation between soil environmental factors and soil microbial community structure diversity under the triple-cropping system in the double-cropping rice area of the middle reaches of the Yangtze River were studied by 16S rRNA high-throughput sequencing and real-time fluorescence quantitative polymerase chain reaction (PCR). The results showed that after two years of experiment, the pH values of each treatment increased, and the rapeseed–early rice–late rice (RRR) model performed better. The soil organic matter and total nitrogen content of the milk vetch–early rice–sweet potato || late soybean (CRI) model was the highest, which increased by 7.89~35.02% and 6.59~26.80% compared with other treatments. The content of soil available phosphorus and available potassium in the potato–early rice–late rice (PRR) model was higher than that in other treatments, which was increased by 29.48% and 126.49% compared with the control. The Chinese milk vetch–early rice–sweet potato || late soybean (CRI) and rapeseed–early rice–sweet potato || late soybean (RRI) models were beneficial to increasing soil nitrate nitrogen and ammonium nitrogen content. Chinese milk vetch–early rice–sweet potato || late soybean (CRI) and rapeseed–early rice–late rice (RRR) patterns were beneficial for improving the microbial diversity index. *Proteobacteria*, *Chloroflexi*, and *Actinobacteria* are the top three dominant phyla in terms of the relative abundance of soil bacteria, and the top three dominant fungi are *Ascomycota*, *Basidiomycota*, and *Mucor*. The Chinese milk vetch–early rice–sweet potato || late soybean (CRI) and rapeseed–early rice–sweet potato || late soybean (RRI) patterns increased the relative abundance of soil *Actinobacteria* and *Ascomycota*. The contents of ammonium nitrogen, total organic carbon, nitrate nitrogen, and available phosphorus were the main environmental factors affecting soil microbial community structure. The findings can provide references for screening out the planting patterns suitable for the promotion of double-cropping rice areas in the middle reaches of the Yangtze River.

## 1. Introduction

Soil microorganisms are one of the important components of the soil ecosystem, which plays an important role in the formation of soil humus, the decomposition of organic matter, and nutrient cycling and transformation [1,2]. Soil microorganisms are not only the driving force of soil nutrient cycling and transformation but also affect soil structure, the soil environment, and crop growth and health [3,4]. They are the functional embodiment of soil and the index of environmental change and maintain soil health and fertility [5]. They are key drivers of nutrient cycling, decomposition of organic matter, and suppression of diseases [6]. Microbial diversity is vital for the sustainability of the agricultural system and the production of healthy crops. Complex soil environmental and agricultural practices affect the diversity and composition of microbes. Nevertheless, microbial diversity is seriously threatened due to rapid climate change, intensive agricultural practices, and land use change [7]. The loss in microbial diversity negatively affects nutrient cycling and disease resistance. which are essential for achieving better crop productivity [8].

Agricultural practices, including, tillage, the use of fertilizers, crop rotation and cover cropping, affect the diversity and composition of soil microbes [9,10,11,12]. Crop rotation is an effective practice which increases the number of soil bacteria and rhizosphere soil bacteria [13]. Studies have shown that there are more soil microorganisms in rotation cropping than that in continuous cropping [14]. Different rotation methods have different effects on microorganisms [15]. Zhang et al. [16] found that the long-term incorporation of green manure in winter could change the microbial community structure of paddy soil and improve microbial diversity and activity. Green manure incorporation could also increase microbial richness [17], increase the proportion of Bacillus and Proteus [18], and have a significant effect on the relative abundance of dominant phyla such as *Proteobacteria*, *Acidobacteria*, *Gemmatimonadetes* and *Nitrobacteria* [19]. Ding et al. [20] showed that the rice–turtle symbiotic system was beneficial in improving the relative abundance of the dominant bacterial genus, improving the diversity and activity of the bacterial community in paddy soil, stabilizing the soil microenvironment and improving soil fertility. Hu et al. [21] found that straw returning could increase the Chao1 richness index and Shannon diversity index of the rice rhizosphere bacterial community, which increased by 14.42% and 4.39%, respectively. There are few studies which have been conducted to determine the impact of different cropping patterns (CRR, CRI, RRR, RRI, and PRR) on the diversity and microbial community structure of paddy fields in the middle reaches of the Yangtze River under the triple-cropping system. Therefore, this study was conducted, using the methods of field experiments, 16S rRNA high-throughput sequencing and real-time fluorescence quantitative polymerase chain reaction (PCR).

Rice is a major cropping system of China and promoting the appropriate rice farming system, and improving the multiple-cropping index, are effective measures for alleviating the food crisis [22]. The middle reaches of the Yangtze River are an important area for grain production and it also an important area of double- and triple-rice production in China. At present, there are many problems in the middle reaches of the Yangtze River, such as single-cropping systems, long-term continuous cropping or multiple cropping, reductions in organic fertilizer input, and low-resource utilization rates, which lead to the decline of paddy soil quality [23] and restrict the development of farmland productivity [24]. Promoting the sustainable development of green ecology in its paddy fields by improving soil fertility and soil ecological environment quality is an important measure [25]. Related studies have shown that paddy–upland multiple cropping and winter-planting green manure can conserve water resources, improve water-use efficiency, increase soil nutrient content [26], improve soil structure, accelerate the mineralization and decomposition of soil nutrients, promote the transformation and flow of soil nutrients [27], reduce soil erosion, and reduce harmful gas emissions [28]. Among them, leguminous green manure has better nitrogen fixation capacity [29], which is conducive to reducing fertilizer input, improving the quality of cultivated land, and promoting agricultural green production [30]. Therefore, this study was conducted with the following aims: (1) to deeply analyze the changes in soil chemical properties and microbial community structure diversity; (2) analyze the correlation between soil environmental factors and soil microbial community structure diversity under the triple-cropping system of paddy fields in the middle reaches of the Yangtze River; (3) explore the high-yield and high-efficiency planting modes suitable for the middle reaches of the Yangtze River. This study can provide a reference for screening the planting patterns suitable for the promotion of double-cropping rice areas in the middle reaches of the Yangtze River.

## 2. Results

### 2.1. The Effects of Triple-Cropping System on Soil Chemical Properties in Paddy Fields in the Middle Reaches of the Yangtze River

The pH value of RRR (rapeseed–early rice–late rice) for early rice treatment in 2021 is the highest (Table 1). PRR (potato–early rice–late rice) treatment had the highest available P content and the highest available K content in winter cropping and early and late rice soil. The soil available K content in the early rice stage was significantly higher than that in other treatments by 28.36% to 52.08% (*p* < 0.05). Soil nitrate–nitrogen under CRI treatment reached the maximum at all stages, and was significantly higher during winter cropping and early rice stages than that of other treatments, by 47.56–132.76% and 90.76–649.89% (*p* < 0.05). In 2022, the organic matter content of early and late rice, soil nitrate nitrogen content of early and late rice, and soil ammonium–nitrogen content of late rice were the highest in CRI treatment. The soil ammonium–nitrogen content of late rice was significantly higher—1.26–1.94 times—than that of other treatments, except the RRI treatment (*p* < 0.05). The total soil nitrogen content of the RRI treatment was the highest in winter cropping and early rice, and was significantly higher than that of other treatments by 19.89% to 35.63% and 17.13% to 30.86% (*p* < 0.05), except for the RRR treatment. The soil available phosphorus content of winter cropping and early rice and the soil available potassium content of early and late rice reached the maximum under the PRR treatment.

### 2.2. The Effects of Triple-Cropping System on Soil Microbial Community Diversity in Paddy Fields in the Middle Reaches of the Yangtze River

The soil bacterial Sobs index of PRR (potato–early rice–late rice) was significantly different from that of CRI (milk vetch–early rice–sweet potato || late soybean) and RRI (rapeseed–early rice–sweet potato || late soybean) (*p* < 0.05; Table 2). The Shannon index of soil bacteria for RRR was significantly higher than that for CRI and RRI—by 9.55% and 7.11%, respectively—and the Shannon index of soil fungi was significantly higher than that in CRI and RRI—by 122.73% and 145.00%, respectively (*p* < 0.05). The Simpson index of soil bacteria treated with CRI was significantly different from that of CK and RRR (*p* < 0.05), and the Simpson index of soil fungi treated with RRI was significantly higher than that of other treatments, by 25.37% and −37.70% (*p* < 0.05). The Coverage index of the soil bacteria and fungi in each treatment was not significantly different (*p* > 0.05). Therefore, the CRI, RRR and RRI treatments can improve the diversity of soil microbial communities.

**Figure 1:**
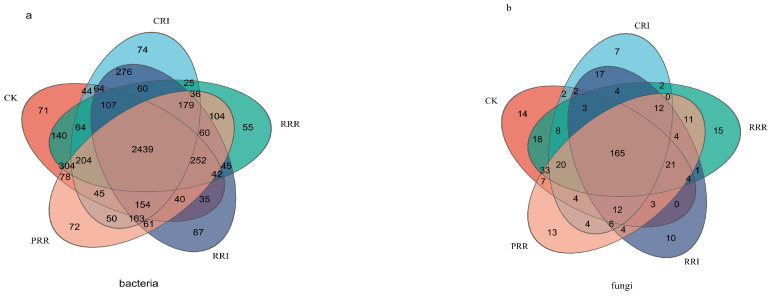
Venn diagram of soil microbial OTU distribution, as affected by different cropping systems. Note: CK: Chinese milk vetch–early rice–late rice; CRI: Chinese milk vetch–early rice–sweet potato || late soybean; RRR: rapeseed–early rice–late rice; RRI: rapeseed–early rice–sweet potato || late soybean; PRR: potato–early rice–late rice. Figure (**a**) is the OTU distribution of bacteria and Figure (**b**) is the OTU distribution of fungi.

The number in the figure represents the number of OTUs in the region (Figure 1). For the treatments CRR, CRI, RRR, RRI and PRR, the soil bacteria contains 4083, 3984, 4116, 4064 and 4241 OUTs, respectively, of which there are 71, 74, 55, 87 and 72 unique OUTs, respectively (Figure 1).

Their soil fungi contained 316, 268, 321, 268 and 319 OUTs, respectively, of which 14, 7, 15, 10 and 13 were unique OUTs.

**Figure 2:**
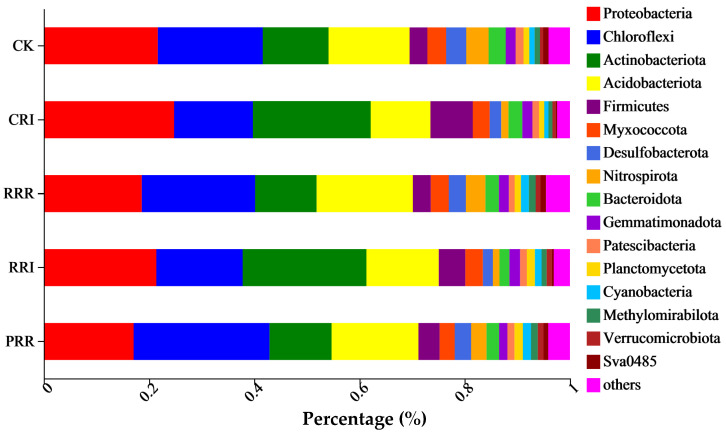
Effect of different cropping systems on dominant species abundance of soil bacterial community at phylum level. Note: CK: Chinese milk vetch–early rice–late rice; CRI: Chinese milk vetch–early rice–sweet potato || late soybean; RRR: rapeseed–early rice–late rice; RRI: rapeseed–early rice–sweet potato || late soybean; PRR: potato–early rice–late rice.

The species composition ratio of different planting patterns at the classification level can reflect the changes in microbial community structure in paddy fields. The top five dominant genera in soil bacteria are *Proteobacteria* (14.98~25.84%), *Chloroflexi* (14.98~25.84%), *Actinobacteriota* (11.36~18.30%), *Acidobacteriota* (11.69~23.51%) and *Firmicutes* (3.37~8.04%) (Figure 2).

**Figure 3:**
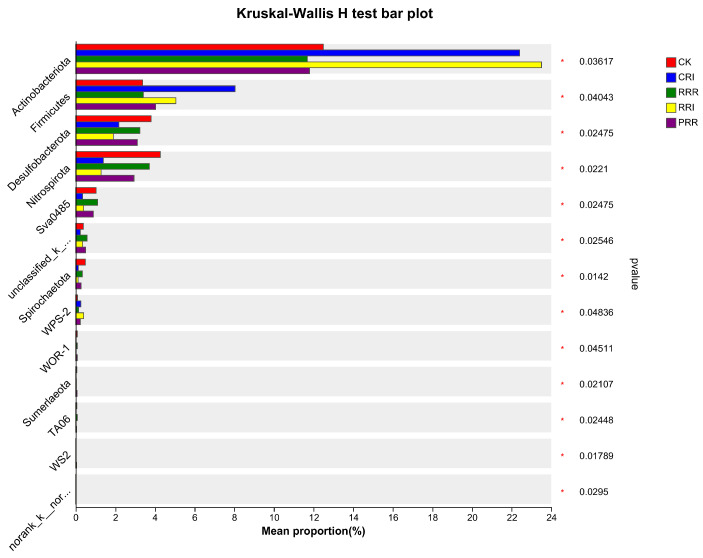
Analysis of inter-group differences in bacterial phylum levels in paddy soil. Note: CK: Chinese milk vetch–early rice–late rice; CRI: Chinese milk vetch–early rice–sweet potato || late soybean; RRR: rapeseed–early rice–late rice; RRI: rapeseed–early rice–sweet potato || late soybean; PRR: potato–early rice–late rice.

The relative abundance of *Actinobacteria* in the RRI treatment was the highest (23.51%), followed by the CRI treatment (22.4%: Figure 3). The relative abundance of *Actinobacteria* in the CRI and RRI treatments was significantly higher than that in other treatments (*p* < 0.05). The highest relative abundance of *Firmicutes* was the treatment CRI, which was 8.04%, followed by the treatment RRI. The relative abundance of *Desulfobacterota* and *Nitrospirota* in CK was the highest, at 3.80% and 4.28%, respectively.

**Figure 4:**
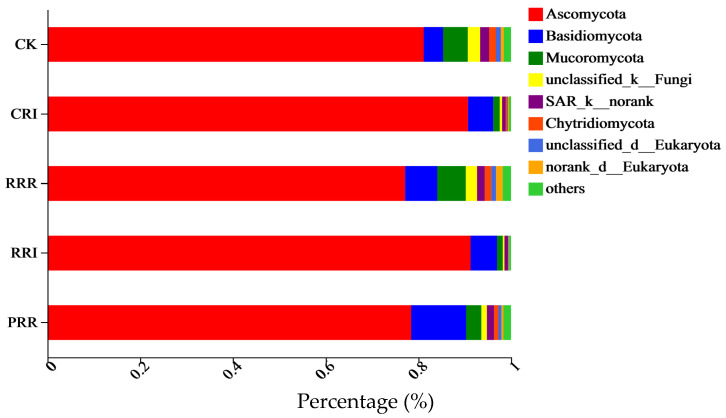
Effect of different cropping systems on abundance of fungal species at phylum level. Note: CK: Chinese milk vetch–early rice–late rice; CRI: Chinese milk vetch–early rice–sweet potato || late soybean; RRR: rapeseed–early rice–late rice; RRI: rapeseed–early rice–sweet potato || late soybean; PRR: potato–early rice–late rice.

Regarding the dominant species abundance of soil fungi at the phylum level, *Ascomycota*, *Basidiomycota* and *Mucoromycota* were the dominant groups. *Ascomycota* accounted for 77.15~91.28%, *Basidiomycota* accounted for 4.16~11.83%, and *Mucoromycota* accounted for 1.24~6.15% (Figure 4).

**Figure 5:**
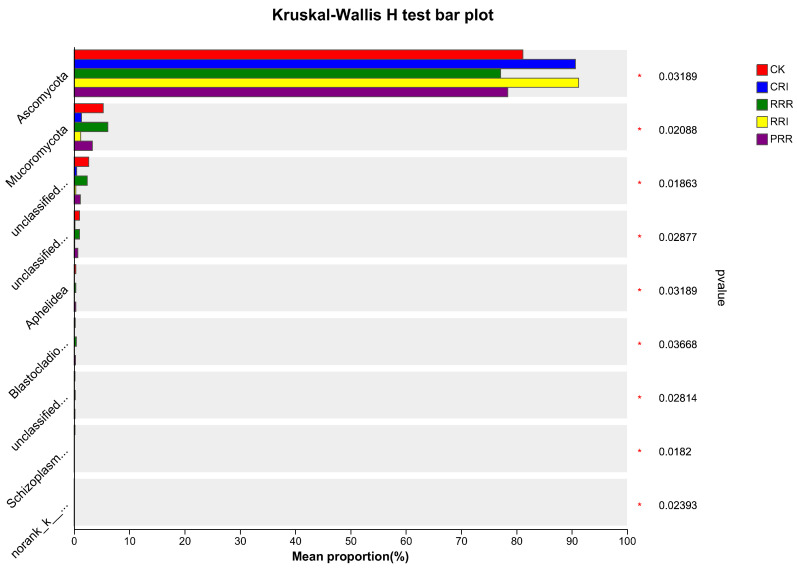
Analysis of inter-group differences at phylum level in paddy soil. Note: CK: Chinese milk vetch–early rice–late rice; CRI: Chinese milk vetch–early rice–sweet potato || late soybean; RRR: rapeseed–early rice–late rice; RRI: rapeseed–early rice–sweet potato || late soybean; PRR: potato–early rice–late rice.

By analyzing the differences between groups of soil fungi at the phylum level (Figure 5), it can be found that the relative abundance of *Ascomycota* in each treatment was significantly different. The relative abundance of *Ascomycota* in CRI and RRI was the highest, which was 90.72% and 91.28%, respectively. The highest relative abundance of *Mucor* was for the RRR treatment, which was 6.15%.

### 2.3. Correlation Analysis Between Soil Environmental Factors and Soil Microbial Community Structure Diversity

**Figure 6:**
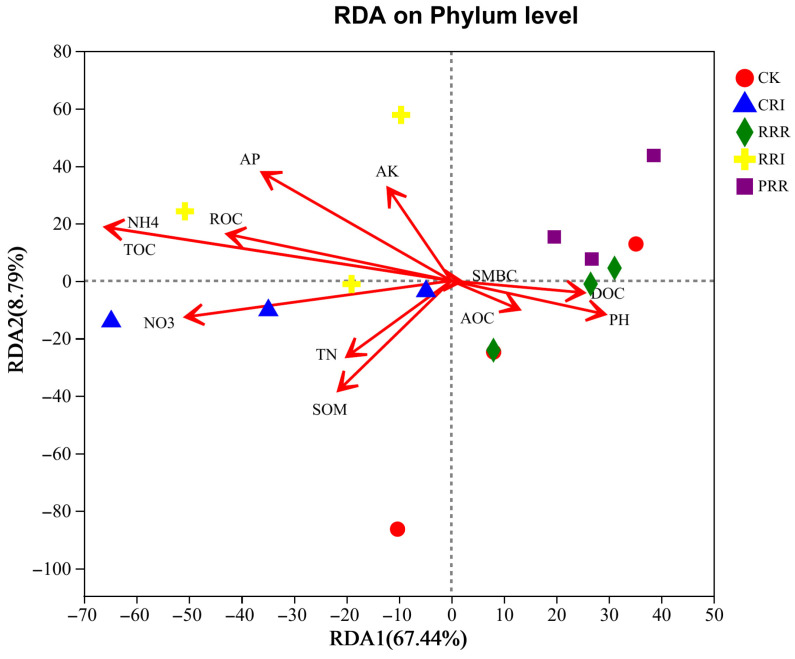
RDA of soil bacterial community structure and soil chemical properties. Note: SOM represents organic matter, TN represents total nitrogen, AP represents available phosphorus, AK represents available potassium, NO_3_ represents nitrate nitrogen, NH_4_ represents ammonium nitrogen, TOC represents soil organic carbon, AOC represents active organic carbon, SMBC represents microbial biomass carbon, DOC represents soluble organic carbon, ROC represents oxidized organic carbon. CK: Chinese milk vetch–early rice–late rice; CRI: Chinese milk vetch–early rice–sweet potato || late soybean; RRR: rapeseed–early rice–late rice; RRI: rapeseed–early rice–sweet potato || late soybean; PRR: potato–early rice–late rice.

The following shows the relative contribution of paddy soil environmental factors to soil bacteria at the phylum level. The results indicated that 67.44% of the variation in soil bacterial community was explained by the first ordination axis (Figure 6). Ammonium nitrogen, total organic carbon, nitrate nitrogen, available phosphorus and easily oxidized organic carbon were the main environmental factors affecting soil bacterial community structure. The CRI treatment was closely related to ammonium nitrogen, total organic carbon, easily oxidized organic carbon, available phosphorus and available potassium, and the treatment RRI was more closely related to nitrate nitrogen, total nitrogen, and organic matter.

**Figure 7:**
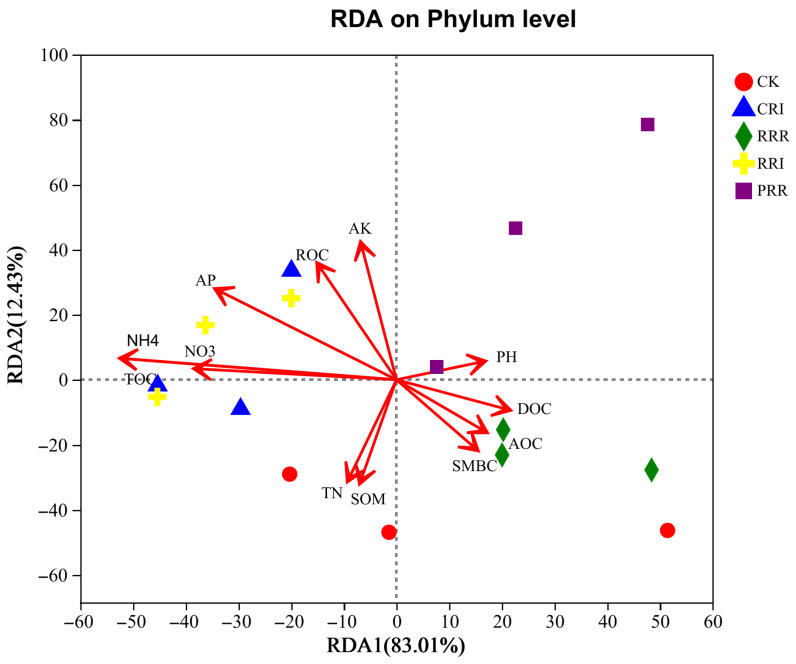
RDA of soil fungal community structure and soil chemical properties. Note: SOM represents organic matter, TN represents total nitrogen, AP represents available phosphorus, AK represents available potassium, NO3 represents nitrate nitrogen, NH4 represents ammonium nitrogen, TOC represents soil organic carbon, AOC represents active organic carbon, SMBC represents microbial biomass carbon, DOC represents soluble organic carbon, ROC represents oxidized organic carbon. CK: Chinese milk vetch–early rice–late rice; CRI: Chinese milk vetch–early rice–sweet potato || late soybean; RRR: rapeseed–early rice–late rice; RRI: rapeseed–early rice–sweet potato || late soybean; PRR: potato–early rice–late rice.

The results indicated an 83.01% variation in the soil fungal community was explained by the first ordination axis (Figure 7). Nitrate nitrogen, ammonium nitrogen and total organic carbon were the main environmental factors affecting soil fungal community structure. Soil nitrate nitrogen, total organic carbon, ammonium nitrogen, available phosphorus, available potassium and readily oxidizable organic carbon were positively related to CRI and RRI. Soil microbial biomass carbon, soluble organic carbon and active organic carbon were more closely related to RRR.

**Figure 8:**
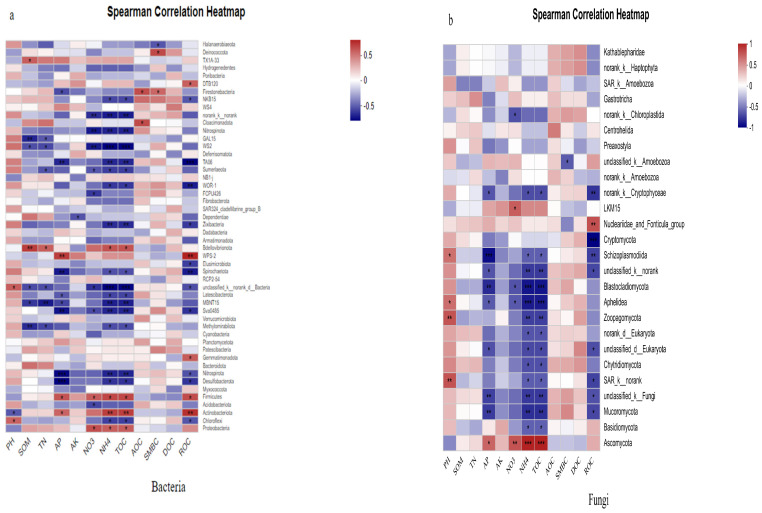
Heatmap of correlation between soil environmental factors and microbial communities (phylum level). Note: CK: Chinese milk vetch–early rice–late rice; CRI: Chinese milk vetch–early rice–sweet potato || late soybean; RRR: rapeseed–early rice–late rice; RRI: rapeseed–early rice–sweet potato || late soybean; PRR: potato–early rice–late rice. SOM: soil organic matter; TN: total nitrogen; AP: available phosphorus; AK: available potassium; NO3: NO_3_-N; NH4: NH_4_^+^-N; AOC: active organic carbon; SMBC: microbial biomass carbon; DOC: dissolved organic carbon; ROC: particulate organic carbon. Figure (**a**) is the spearman correlation heatmap of bacteria, and Figure (**b**) is the spearman correlation heatmap of fungi. *, ** and *** stand for *p* < 0.05, *p* < 0.01 and *p* < 0.001 respectively.

As shown in the heat map of the correlation between soil environmental factors and microbial communities (Figure 8), organic matter in soil bacteria is significantly positively correlated with the relative abundance of *Bdellovibrionota*, and significantly negatively correlated with the relative abundance of *Methylomirabilota* and *GAL15*. Nitrate nitrogen, ammonium nitrogen and total organic carbon were significantly negatively correlated with the relative abundance of *norank _ k _ norank*, *Nitrospirota* and *WS2*. Ammonium nitrogen and total organic carbon were also significantly negatively correlated with the relative abundance of *unclassified _ k _ norank _ d _ Bacteria*, and significantly positively correlated with the relative abundance of *Actinobacteriota*. The readily oxidizable organic carbon was significantly positively correlated with the relative abundance of *WPS-2* and *Actinobacteria*, and significantly negatively correlated with the relative abundance of *TA06*, *WOR-1* and *Spirochaetota*.

As for fungi, pH was significantly positively correlated with the relative abundance of *Zoopagomycota* and *SAR_k_norank*. Available phosphorus was significantly negatively correlated with the relative abundance of *Schizoplasmodiida*, *Blastocladiomycota*, *unclassified_k_Fungi* and *Mucoromycota*. Nitrate nitrogen, ammonium nitrogen and total organic carbon were significantly positively correlated with the relative abundance of *Ascomycota*. However, ammonium nitrogen and total organic carbon were significantly negatively correlated with the relative abundance of *Cladosporium* and *Aphelidea*. The oxidizable organic carbon was significantly positively correlated with the relative abundance of the *Nucleariidae* and *Fonticula* groups, but significantly negatively correlated with the relative abundance of *Cryptomycota*, *norank _k_ Cryptophyceae* and *Schizoplasmodiida*.

## 3. Discussions

### 3.1. Effects of Triple-Cropping System on Soil Chemical Properties in Double-Cropping Rice Area of Middle Reaches of Yangtze River

Compared with the continuous cropping mode, paddy–upland multiple cropping can improve soil fertility. Ji et al. [31] showed that the total nitrogen and organic matter content of soil treated with milk vetch instead of chemical fertilizer increased significantly. Wan et al. [32] showed that the total nitrogen and organic matter content of soil applied with milk vetch green manure were higher than those of pure chemical fertilizer. The results of this study showed that after two years of experiments, the soil pH value of each treatment increased to a certain extent. Winter planting of green manure could increase soil pH value, alleviate soil acidification and improve soil conditions. At the 2021 early rice stage, the RRR treatment had a significantly higher pH (4.97 ± 0.09) compared to CRI (4.60 ± 0.11) and PRR (4.89 ± 0.07). This is because rapeseed cultivation promotes nitrifying bacteria activity, converting ammonium nitrogen (NH_4_^+^) into nitrate nitrogen (NO_3_^−^), which releases OH^−^ and raises soil pH. In contrast, other cropping systems (e.g., Chinese milk vetch–early rice) may exacerbate acidification due to H^+^ secretion from leguminous nitrogen fixation. In this study, the soil organic matter content of the Chinese milk vetch–early rice–sweet potato || late soybean (CRI) model performed better than the control. This may be because green manure can increase the organic matter in the soil.

At the same time, multiple cropping can improve soil permeability, enhance soil permeability and improve fertilizer utilization. Wang et al. [33] showed that Chinese milk vetch can effectively improve the total nitrogen content of paddy soil. This study showed that after the late rice harvest in 2022, the soil organic matter and total nitrogen content of the Chinese milk vetch–early rice–sweet potato || late soybean (CRI) model were the highest, with an increase of 7.89~35.02% and 6.59~26.80% compared with other treatments. The likely reason is that the sufficient nutrients in the soil after winter crop incorporation promoted microbial proliferation, and microbial activity facilitates the decomposition and release of organic nutrients while increasing soil organic matter content. At the same time, soybeans can fix solid nitrogen in the air through rhizobia and increase soil total nitrogen content. Based on the two-year data, compared with the control, the soil available phosphorus and soil available potassium contents in the potato–early rice–late rice (PRR) model were higher than those in other treatments. This may be due to the slow release of soil nutrients under flooding conditions, and the soil colloid adsorbs a large amount of potassium and phosphorus. In addition, potato residues have a high carbon-to-nitrogen ratio (C/N), resulting in slow decomposition and the gradual release of mineral nutrients such as phosphorus and potassium. Root exudates (e.g., organic acids) activate insoluble phosphorus in the soil, increasing available phosphorus content. Liu et al. [34] showed that the green manure of *Astragalus sinicus* could increase the content of available nitrogen in soil. Zhang et al. [35] showed that paddy–upland rotation could increase nitrogen accumulation in paddy soil. The results of this study showed that CRI and RRI could significantly increase the content of nitrate nitrogen and ammonium nitrogen in soil. The amount of nitrogen fertilizer applied to dry crops was larger, and the application of nitrogen fertilizer promoted the increase in soil nitrogen content. Nitrogen loss is serious in the early rice season and winter fallow season in paddy fields [36], while soybean has a strong nitrogen fixation ability in dry crops, which can slow down nitrogen loss in soil.

### 3.2. Effects of Triple-Cropping System on Soil Microbial Community Structure Diversity in Double-Cropping Rice Paddy Field in Middle Reaches of Yangtze River

#### 3.2.1. Soil Microbial α Diversity

Soil microorganisms can promote the decomposition and transformation of soil nutrients, form a good soil structure, and promote crop growth. Liu et al. [37] found that straw returning can provide exogenous organic materials for bacteria, promote the growth and reproduction of bacteria [38], and improve the diversity of soil microorganisms. Green manure incorporation can increase the number of bacteria and fungi in soil [39], because green manure incorporation can release a large amount of nutrients, improve the microbial characteristics of paddy soil, and affect the relative abundance of soil bacteria and fungi [40]. Jin et al. [41] found that straw returning could increase the diversity index of soil fungi in rice–oilseed rape rotation. The results of this experiment showed that paddy–upland multiple cropping had a certain effect on the diversity of soil bacteria and fungi.

The Chinese milk vetch–early rice–sweet potato || late soybean (CRI) and rapeseed–early rice–sweet potato || late soybean (RRI) models could improve the Simpson index of soil bacteria and fungi. The rapeseed–early rice–late rice (RRR) model could improve the Sobs index and Shannon diversity index of soil bacteria and fungi. The bacterial Alpha diversity in the paddy field was higher than that in dry land [42], because the paddy field was flooded to form an anaerobic environment, and the anaerobic environment could enhance the stability between soil bacteria [43], thus improving bacterial diversity.

#### 3.2.2. Soil Microbial Species Composition

Straw returning can increase the relative abundance of soil *Proteobacteria* and *Chloroflexi* [44]. Pu et al. [45] showed that the dominant genus groups in paddy soil were mainly *Chloroflexi*, *Proteobacteria* and *Actinobacteria*. Green manure turnover can increase the relative abundance of soil *Proteobacteria* and *Actinobacteria*. Lin et al. [46] found that the dominant groups of paddy soil in the early and late rice season were *Proteobacteria*, *Nitrospira*, *Acidobacteria* and *Bacteroidetes*. The results of this research showed that the dominant genera in soil bacteria were *Proteobacteria* (17.05~24.76%), *Chloroflexi* (14.98~25.84%) and *Actinobacteria* (11.69~23.51%). *Proteobacteria* are involved in soil nutrient cycling and can better ensure soil fertility [47]. *Chloroflexi* can ferment sugars and polysaccharides and promote the decomposition of organic matter in rice soil [47]. *Actinobacteria* can accelerate the decay of animal and plant remains in soil, which plays an important role in accelerating soil material circulation and energy flow and the construction of the soil environment [48]. The results of this experiment showed that the relative abundance of *Actinobacteria* in the Chinese milk vetch–early rice–sweet potato || late soybean (CRI) model and rapeseed–early rice–sweet potato | late soybean RRI) model was significantly higher than that of other treatments. The enrichment of *Actinobacteria* and *Firmicutes* in CRI and RRI treatments results from the combined effects of residue chemical composition, alternating flooded–upland habitats, and microbial functional strategies. Chinese milk vetch’s low C/N residues trigger a short-term proliferation of *Firmicutes*, whereas rapeseed’s high C/N residues and lignin content sustain the long-term dominance of *Actinobacteria*. These differences in microbial community structure reflect how distinct cropping patterns regulate soil carbon and nitrogen cycling pathways, providing a theoretical basis for optimizing paddy field management [49].

The return of Chinese milk vetch, rapeseed and green manure can provide a large amount of humus, and the organic carbon of paddy–upland multiple cropping treatment is higher than that of other treatments, which provides sufficient growth conditions for actinomycetes. The dominant fungal groups in this study were *Ascomycota*, *Basidiomycota* and *Mucoromycota. Ascomycota* accounted for 77.15~91.28%, *Basidiomycota* accounted for 4.16~11.83% and *Mucoromycota* accounted for 1.24~6.15%.

The relative abundance of *Ascomycota* in each treatment was significantly different. The relative abundance of *Ascomycota* in CRI and RRI was the highest, which was 90.72% and 91.28%, respectively. This is also consistent with Nie et al. [50], which stated that soil fungi are mainly composed of *Ascomycota* and *Basidiomycota*. Xu et al. [51] also showed that *Ascomycota* and *Basidiomycota* were the dominant phyla in soil fungi. In this experiment, the relative abundance of *Ascomycota* in CRI and RRI was the highest, which may be because water content, as a key factor, affected the structure of the soil fungal community [52,53].

### 3.3. Correlation Analysis Between Soil Environmental Factors and Soil Microorganisms

Soil microorganisms are closely related to soil fertility. The community structure of soil fungi and bacteria profoundly affects the physical and chemical properties of soil, and the physical and chemical properties of soil can affect soil microorganisms in turn. The results of this experiment showed that ammonium nitrogen, total organic carbon, nitrate nitrogen and available phosphorus were the main environmental factors affecting soil microbial community structure. Ammonium Nitrogen (NH_4_^+^-N) exhibited a highly significant positive correlation with *Actinobacteria* (r = 0.82, *p* < 0.01), as they utilize NH_4_^+^-N to synthesize extracellular enzymes for organic matter degradation. Total Organic Carbon (TOC) drove the growth of *Chloroflexi* (r = 0.75) and *Proteobacteria* (r = 0.68) by supplying energy substrates. Nitrate Nitrogen (NO_3_^−^-N) enhanced the activity of denitrifying bacteria (e.g., Proteobacteria). The high NO_3_^−^-N in CRI was positively correlated with *Proteobacteria* abundance. Available Phosphorus (AP) showed a negative correlation with *Acidobacteriota* (r = −0.61), as phosphorus limitation reduces their competitive advantage. The CRI treatment, characterized by high TOC, NH_4_^+^-N, and AP (Figure 6), supported the proliferation of *Actinobacteria* (22.4%) and *Proteobacteria* (24.76%), forming a microbial network centered on efficient carbon and nitrogen cycling. There was also a positive link between soil organic matter content and soil microbes, which aligns with earlier results where authors also found a positive association between soil organic matter content and soil microbes [53]. Yang et al. [54] found that the relative abundance of fungal species (*Sarocladium*) in paddy soil was significantly positively correlated with soil total organic carbon. Organic carbon and available nitrogen were the main environmental factors affecting soil fungal community structure [55,56]. Soil organic carbon and available nitrogen can provide the necessary carbon and nitrogen sources for the survival of soil microorganisms and promote the growth of microorganisms. In this experiment, soil ammonium nitrogen and total organic carbon were significantly negatively correlated with the relative abundance of *Cladosporium*, *Aphelidea* and *unclassified _ k _ norank _ d _ Bacteria*. Soil nitrate nitrogen, ammonium nitrogen and total organic carbon were significantly negatively correlated with the relative abundance of *norank _ k _ norank*, *Nitrospirota* and *WS2*, indicating that the growth of these bacteria did not have a high demand for soil carbon and nitrogen. On the other hand, it may also be because these bacteria are inefficient carbon and nitrogen utilization species [57]. Ammonium nitrogen and total organic carbon were significantly positively correlated with the relative abundance of *Actinobacteriota*. Nitrate nitrogen, ammonium nitrogen and total organic carbon were significantly positively correlated with the relative abundance of *Ascomycota*. In the multiple-cropping treatment, the return of dry crop straw to the field increases the input of the carbon source, which is conducive to the growth and reproduction of *Actinomycetes*.

Heatmap analysis reveals significant correlations between microbial taxa and environmental factors. Soil organic carbon (SOC) and available nitrogen (AN) provide essential carbon and nitrogen sources for microbial survival, thereby promoting microbial growth and activity. The relative abundances of *Actinobacteriota* and *Ascomycota* exhibit highly significant positive correlations with soil carbon and nitrogen levels (*p* < 0.01), demonstrating that these nutrients are essential for their growth and proliferation. The paddy–upland rotation system (CRI/RRI) significantly improved the evenness of bacterial and fungal diversity (as indicated by the Simpson index), while the conventional rice–rice rotation (RRR) was more conducive to species richness (reflected in Sobs/Shannon indices). Key findings demonstrate that green manure incorporation increased the relative abundance of *Actinobacteriota* by 23.51% compared to monoculture systems, with their saprophytic characteristics enabling efficient decomposition of green manure humus. Concurrently, the high organic carbon environment promoted Ascomycota dominance, reaching 91.28% of the fungal community. The carbon–nitrogen synergy analysis revealed that total organic carbon (TOC) and ammonium nitrogen (NH_4_^+^-N) significantly stimulated the proliferation of *Actinobacteriota* (+35%) and *Ascomycota* (+28%), while simultaneously suppressing *Blastocladiomycota* (−42%) and *Nitrospirota* (−38%). These results elucidate the differential regulatory mechanisms of microbial carbon and nitrogen utilization efficiency. These microbial community changes facilitate the establishment of an efficient nutrient cycling network through *Proteobacteria*-mediated polysaccharide decomposition and *Chloroflexi*-driven organic matter mineralization, ultimately enhancing the sustainability of soil fertility. The negative correlation between microbial communities and soil nutrients reflects the ecosystem’s response to high-input agricultural practices. Over the long term, these relationships may compromise soil self-sustaining capacity, leading to increased resource waste and environmental risks. By optimizing fertilization practices, improving soil health, and preserving microbial diversity, we can restore beneficial microbe–nutrient interactions to achieve sustainable soil utilization.

## 4. Materials and Methods

### 4.1. Experimental Site

The experiment was carried out in the rice experimental field of Jiangxi Agricultural University Science and Technology Park from 2020 to 2022. The area belongs to a subtropical humid monsoon climate. The average annual total solar radiation is 4.79 * 10^13^ J hm^−2^, the average annual sunshine hours are 1532.9 h, the average annual temperature is 19.4 °C, and the average annual precipitation is 2051.1 mm. Before the experiment, the pH value of 0~15 cm soil was 5.28, the organic matter was 28.48 g kg^−1^, the total nitrogen was 1.99 g kg^−1^, the available phosphorus was 28.99 mg kg^−1^, and the available potassium was 19.07 mg kg^−1^.

### 4.2. Test Materials and Field Experiment Design

This experiment was a field experiment with a randomized block design. A total of 5 treatments were designed, which were Chinese milk vetch–early rice–late rice (CRR), Chinese milk vetch–early rice–sweet potato || late soybean (CRI), rapeseed–early rice–late rice (RRR), rapeseed–early rice–sweet potato || late soybean (RRI) and potato–early rice–late rice (PRR) (Table 3). Each treatment had three replicates and a total of 15 plots. The area of each plot was 33 m^2^ (11 m × 3 m), and the soil fertility of each plot was the same before the experiment.

Chinese milk vetch and rapeseed were sown, and potato was cut and planted. The sowing rate of Chinese milk vetch was 37.5 kg/hm^2^, the sowing rate of rapeseed was 15 kg/hm^2^, and the planting density of potatoes was 73,000 plants/hm^2^. Fifteen days before rice transplanting, the straws of milk vetch, rapeseed and potato were all turned back to the field. The sweet potato and soybean were furrowed and ridged, with a ridge width of 1.2 m and a ridge height of 0.35 m. Each ridge was planted with 4 rows of soybean and 1 row of sweet potato. Sweet potato was sowed in strips. The row spacing between sweet potato was 0.3 m, the plant spacing was 0.25 m, and the planting density was 18,182 plants/hm^2^. With hole sowing, soybean row spacing was 0.2 m. Plant spacing was 0.2 m, and there was a planting density of 145,455 plants/hm^2^. The row spacing of rice is 0.2 m, and the plant spacing is 0.2 m. The specific field management is shown in Table 4.

### 4.3. Determination of Soil Chemical Properties

Soil samples from the 0–20 cm plow layer were collected at each point with a ‘5-point sampling method’ at the maturity stage of winter crops and after harvest of early and late rice, respectively. Specifically, the intersection point of the field diagonals was designated as the central sampling location. Four equidistant sampling points were established along the diagonals radiating from this center. The soil samples of each plot were mixed evenly. In total, 15 samples were obtained for each stage. One part was naturally air-dried to determine the content of soil pH, soil organic matter, total nitrogen, available phosphorus, available potassium [58,59] and total organic carbon, and the other part was sealed in a 4 °C (<72 h) refrigerator to determine active organic carbon, dissolved organic carbon, particulate organic carbon and microbial biomass carbon [60,61,62].

### 4.4. Determination of Soil Microbial Community Structure Diversity

After the late rice was harvested, the 0~20 cm topsoil was randomly taken from each plot by the ‘five-point sampling method’. After mixing, it was immediately frozen in liquid nitrogen and stored in a refrigerator at −80 °C. The sample was commissioned by Meiji Biomedical Technology Co., Ltd. for high-throughput sequencing. The main steps are as follows: First, the microbial DNA in the soil sample was extracted using the DNeasy^®^ PowerSoil^®^ Pro Kit from QIAGEN, Germantown, MD, USA, and then the DNA concentration and purity were determined by an ultra-micro spectrophotometer (Beijing Persee General Instrument Co., Ltd., Beijing, China), and DNA integrity was detected by 1% agarose gel electrophoresis. Primer 338F: ACTCCTACGGGGAGGCAGCAG and primer 806R: GGACTACHVGGGTWTCTAAT were used to amplify the 16S rRNA of microorganisms [63]. Primer SSU0817: FTTAGCATGGAATAATRRAATAGGA and primer 1196R: TCTGGACCTGGTGAGTTTCC were used to amplify the 18sDNA of microorganisms. The products were detected by 2% agarose gel electrophoresis. The PCR products were identified, purified and quantified, and the Miseq library was constructed. Sequencing was performed using the Illumina Miseq PE300 platform (commissioned by Shanghai, Meiji Biotechnology Co., Ltd., Shanghai, China).

### 4.5. Data Processing

FLASH v1.2.7 software was used to splice the number of double-ended reads of soil bacteria obtained by high-throughput sequencing, and then Qiimev1.9.1 software was used to process the final valid tag results. With Up-arsev7.0.1001 software, the final effective data results, with a 97% similarity, were aggregated to obtain an operational classification unit (OTU). The α-diversity index, including the Chao1 index, Shannon index, and species number were calculated by Mothur polymerization results for the OTU. Chao1 is suitable for assessing changes in total species richness, while Shannon index captures overall differences in community structure. The combined use of multiple indices avoids the limitations of relying on a single metric, thereby providing a more comprehensive understanding of the ecological significance of microbial communities.

The data were processed by Microsoft Excel 2019, and the data statistics and variance analysis were performed by SPSS 22.0 system software. One-way ANOVA and the Duncan method were used for variance analysis and multiple comparisons. LSD was used to compare the difference in sample averages. Origin 8.0 and R Studio (4.2.0) were used for making figures. The Pearson correlation coefficient and redundancy analysis (RDA) were performed to determine the correlation between soil properties and soil microbial population composition. The analysis of soil microbial community structure was carried out by using the cloud platform of Shanghai Meiji Biological Company (Shanghai, China).

## 5. Conclusions

In conclusion, milk vetch–early rice–sweet potato || late soybean (CRI) and rapeseed–early rice–sweet potato || late soybean (RRI) models were beneficial in increasing soil nitrate nitrogen and ammonium nitrogen content. On the other hand, Chinese milk vetch–early rice–sweet potato || late soybean (CRI) and rapeseed–early rice–late rice (RRR) patterns were beneficial in improving the microbial diversity index. Chinese milk vetch–early rice–sweet potato || late soybean (CRI) and rapeseed–early rice–sweet potato || late soybean (RRI) patterns increased the relative abundance of *Actinobacteria* and *Ascomycota* in soil. Soil ammonium nitrogen, total organic carbon, nitrate nitrogen and available phosphorus were the main environmental factors affecting soil microbial community structure in different cropping patterns. Nevertheless, in the future, the response of a microbial community to dynamic changes of carbon and nitrogen in different planting patterns may be further improved, and the microbial community structure related to nitrogen cycle should be studied. Further, the role of different bacterial and fungal communities in soil organic carbon accumulation in different cropping patterns must also be studied. Additionally, studies on functional metagenomic linking with soil microbes, root metabolites and soil inputs are also needed, and can better our understanding of the impact of different cropping patterns on soil bacterial and fungal communities.

## Figures and Tables

**Table 1:** Soil chemical properties under different planting patterns.

Year	Treatment	Period	pH	Organic Matter (g kg^−1^)	Total N (g kg^−1^)	Available P (mg kg^−1^)	Available K (mg kg^−1^)	NO_3_-N (mg kg^−1^)	NH_4_^+^-N (mg kg^−1^)
2021		Winter crop	5.10 ± 0.04 a	29.38 ± 1.44 a	1.94 ± 0.09 a	37.01 ± 3.07 ab	20.00 ± 2.52 a	30.25 ± 1.83 b	15.50 ± 1.59 a
	CRR(CK)	Early rice	4.78 ± 0.14 ab	31.66 ± 1.96 a	1.88 ± 0.10 a	36.31 ± 2.65 ab	46.67 ± 2.33 b	17.10 ± 3.50 b	9.97 ± 1.47 a
		Late rice	5.78 ± 0.06 a	27.51 ± 1.12 a	1.81 ± 0.10 a	22.84 ± 2.62 a	28.33 ± 4.18 a	13.30 ± 3.50 b	10.63 ± 2.11 a
		Winter crop	5.20 ± 0.15 a	29.83 ± 0.93 a	1.88 ± 0.06 a	36.88 ± 2.59 ab	19.33 ± 2.91 a	48.46 ± 1.69 a	13.01 ± 3.24 a
	CRI	Early rice	4.60 ± 0.11 b	32.12 ± 1.97 a	1.93 ± 0.11 a	29.97 ± 4.81 b	42.67 ± 2.67 b	32.62 ± 6.21 a	7.59 ± 1.14 a
		Late rice	5.61 ± 0.07 a	26.96 ± 0.94 a	1.97 ± 0.12 a	20.52 ± 1.34 a	31.00 ± 3.06 a	27.12 ± 4.37 a	12.75 ± 2.31 a
		Winter crop	4.85 ± 0.10 a	27.93 ± 1.99 a	1.79 ± 0.11 a	33.56 ± 2.81 ab	21.67 ± 4.33 a	32.84 ± 3.06 b	16.00 ± 0.46 a
	RRR	Early rice	4.97 ± 0.09 a	28.96 ± 2.27 a	1.68 ± 0.14 a	31.90 ± 1.21 ab	39.67 ± 1.86 b	6.39 ± 0.22 c	10.24 ± 2.55 a
		Late rice	5.82 ± 0.04 a	27.00 ± 0.46 a	1.69 ± 0.10 a	21.48 ± 1.68 a	28.33 ± 1.86 a	14.53 ± 1.98 ab	11.28 ± 4.90 a
		Winter crop	4.89 ± 0.10 a	27.84 ± 1.39 a	1.77 ± 0.07 a	30.10 ± 3.08 b	14.67 ± 1.45 a	21.15 ± 1.19 c	16.93 ± 3.08 a
	RRI	Early rice	4.70 ± 0.11 ab	29.92 ± 1.78 a	1.79 ± 0.11 a	32.29 ± 4.94 ab	47.00 ± 3.00 b	5.03 ± 1.02 c	13.53 ± 3.70 a
		Late rice	5.65 ± 0.08 a	28.34 ± 0.58 a	1.92 ± 0.08 a	26.65 ± 7.76 a	32.67 ± 5.04 a	19.61 ± 6.76 ab	10.62 ± 3.12 a
		Winter crop	4.81 ± 0.14 a	28.50 ± 1.72 a	1.78 ± 0.14 a	41.65 ± 1.11 a	23.00 ± 2.31 a	20.82 ± 3.17 c	14.57 ± 2.51 a
	PRR	Early rice	4.89 ± 0.07 ab	32.23 ± 1.48 a	1.75 ± 0.18 a	42.96 ± 1.84 a	60.33 ± 5.24 a	4.35 ± 0.39 c	6.91 ± 1.71 a
		Late rice	5.77 ± 0.11 a	26.57 ± 1.08 a	1.62 ± 0.18 a	26.16 ± 0.45 a	36.67 ± 2.19 a	9.37 ± 1.58 b	6.23 ± 1.37 a
2022		Winter crop	5.18 ± 0.13 a	34.36 ± 0.62 a	1.60 ± 0.06 c	28.18 ± 1.11 b	31.00 ± 0.58 a	3.29 ± 0.61 b	18.40 ± 1.31 ab
	CRR (CK)	Early rice	5.13 ± 0.12 a	26.92 ± 1.12 c	1.62 ± 0.07 c	29.32 ± 1.38 bc	33.67 ± 2.03 c	11.23 ± 1.60 b	19.26 ± 2.24 a
		Late rice	5.36 ± 0.02 a	32.10 ± 2.32 ab	1.82 ± 0.15 ab	25.81 ± 0.38 d	27.67 ± 2.60 c	14.00 ± 1.59 b	10.92 ± 1.21 b
		Winter crop	5.38 ± 0.12 a	29.34 ± 0.26 b	1.73 ± 0.01 bc	31.37 ± 0.95 b	32.67 ± 3.28 a	9.82 ± 0.42 a	18.54 ± 1.03 ab
	CRI	Early rice	5.17 ± 0.07 a	33.37 ± 1.52 a	1.81 ± 0.04 bc	31.32 ± 1.69 bc	51.67 ± 1.76 b	22.55 ± 3.24 a	17.86 ± 0.27 a
		Late rice	5.20 ± 0.12 a	34.66 ± 1.38 a	1.94 ± 0.03 a	39.55 ± 1.64 a	52.33 ± 4.33 a	24.80 ± 0.11 a	23.48 ± 1.86 a
		Winter crop	5.40 ± 0.10 a	35.04 ± 1.06 a	1.95 ± 0.05 ab	28.92 ± 1.66 b	32.00 ± 0.58 a	3.48 ± 0.16 b	19.68 ± 1.29 a
	RRR	Early rice	5.27 ± 0.02 a	31.50 ± 1.48 ab	1.96 ± 0.05 ab	27.78 ± 0.70 c	41.00 ± 2.65 c	10.62 ± 2.31 b	19.90 ± 1.15 a
		Late rice	5.14 ± 0.23 a	29.78 ± 1.55 abc	1.76 ± 0.10 ab	30.45 ± 1.62 cd	29.67 ± 2.33 bc	9.83 ± 1.18 c	9.08 ± 0.57 b
		Winter crop	5.32 ± 0.08 a	28.01 ± 1.51 b	2.17 ± 0.08 a	29.22 ± 2.82 b	31.67 ± 2.91 a	2.47 ± 0.26 b	13.67 ± 0.86 c
	RRI	Early rice	5.21 ± 0.06 a	27.24 ± 0.88 c	2.12 ± 0.10 a	33.25 ± 1.75 b	41.00 ± 2.89 c	18.86 ± 2.46 a	7.92 ± 1.24 b
		Late rice	5.04 ± 0.17 a	27.10 ± 0.52 bc	1.69 ± 0.10 ab	35.48 ± 0.60 ab	39.33 ± 4.26 b	14.22 ± 1.47 b	21.03 ± 1.79 a
		Winter crop	5.17 ± 0.04 a	31.60 ± 2.10 ab	1.81 ± 0.11 bc	38.15 ± 1.49 a	34.33 ± 1.86 a	9.56 ± 0.78 a	14.97 ± 1.23 bc
	PRR	Early rice	5.20 ± 0.10 a	28.58 ± 1.26 bc	1.75 ± 0.13 bc	41.12 ± 1.30 a	63.00 ± 4.16 a	17.78 ± 1.18 ab	9.31 ± 1.29 b
		Late rice	5.45 ± 0.03 a	25.67 ± 1.78 c	1.53 ± 0.09 b	33.42 ± 2.31 bc	62.67 ± 2.96 a	10.29 ± 1.35 bc	8.00 ± 0.24 b

Note: Values are means ± standard errors (n = 3). The different lowercase letters indicate significant differences in the variable means among treatments at *p* < 0.05.

**Table 2:** Effect of different cropping systems on diversity index of microbial community.

Microorganism	Treatment	Alpha Diversity Index of Species			
		Sobs	Shannon	Simpson	Coverage
Bacteria	CRR(CK)	45.67 ± 0.33 a	2.38 ± 0.01 a	0.14 ± 0.00 b	1.00 ± 0.00 a
	CRI	42.00 ± 1.53 b	2.20 ± 0.06 b	0.16 ± 0.01 a	1.00 ± 0.00 a
	RRR	45.67 ± 0.67 a	2.41 ± 0.00 a	0.14 ± 0.00 b	1.00 ± 0.00 a
	RRI	42.33 ± 0.33 b	2.25 ± 0.03 b	0.16 ± 0.01 ab	1.00 ± 0.00 a
	PRR	46.67 ± 0.33 a	2.37 ± 0.01 a	0.14 ± 0.01 ab	1.00 ± 0.00 a
Fungi	CRR(CK)	20.67 ± 0.67 a	0.85 ± 0.14 a	0.67 ± 0.07 b	1.00 ± 0.00 a
	CRI	19.00 ± 0.58 a	0.44 ± 0.06 b	0.83 ± 0.03 a	1.00 ± 0.00 a
	RRR	20.33 ± 1.33 a	0.98 ± 0.04 a	0.61 ± 0.03 b	1.00 ± 0.00 a
	RRI	18.33 ± 0.67 a	0.40 ± 0.06 b	0.84 ± 0.03 a	1.00 ± 0.00 a
	PRR	20.00 ± 1.15 a	0.86 ± 0.05 a	0.63 ± 0.03 b	1.00 ± 0.00 a

Note: Values are means ± standard errors (n = 3). Different lowercase letters indicate significant differences in means among treatments at *p* < 0.05 (LSD Test).

**Table 3:** Details of experiment treatments.

Treatment	Cropping Pattern
CRR(CK)	Chinese milk vetch–early rice–late rice
CRI	Chinese milk vetch–early rice–sweet potato || late soybean
RRR	Rapeseed–early rice–late rice (RRR), rapeseed–early rice–sweet potato || late soybean
RRI	Rapeseed–early rice–sweet potato || late soybean
PRR	Potato–early rice–late rice

Note ‘–’ means stubble, ‘||’ means inter-cropping.

**Table 4:** Details of different management practices used during study.

Crop	Variety	Seeding and Harvest Time	Fertilizing Amount
Chinese milk vetch	Yujiang big leaf seed	2.10.2020~6.04.2021 1.10.2021~7.04.2022	No fertilizer
rapeseed	Zhongyou821	4.12.2020~6.04.2021 10.11.2021~7.04.2022	No fertilizer
early rice	Zhongjia early17	2.05.2021~22.07.2021 2.05.2022~23.7.2022	N 180 kg/hm^2^, P_2_O_5_90 kg/hm^2^, K_2_O 120 kg/hm^2^
late rice	Tianyou Huazhan	29.07.2021~24.10.2021 30.07.2022~30.10.2022	N 180 kg/hm^2^, P_2_O_5_90 kg/hm^2^, K_2_O 120 kg/hm^2^
soybean	Fengyuan No.1	13.08.2021~8.11.2021 4.08.2022~18.11.2022	N 150 kg/hm^2^, P_2_O_5_150 kg/hm^2^, K_2_O 375 kg/hm^2^
sweet potato	Guangshu 87	13.08.2021~8.11.2021 4.08.2022~18.11.2022	N 80 kg/hm^2^, P_2_O_5_375 kg/hm^2^, K_2_O 80 kg/hm^2^
potato	Dongnong 303	6.12.2020~10.04.2021 1.12.2021~7.04.2022	No fertilizer

## Data Availability

Data is contained within the article.
